# CeRNA Expression Profiling Identifies KIT-Related circRNA-miRNA-mRNA Networks in Gastrointestinal Stromal Tumour

**DOI:** 10.3389/fgene.2019.00825

**Published:** 2019-09-10

**Authors:** Ning Jia, Hanxing Tong, Yong Zhang, Hiroshi Katayama, Yuan Wang, Weiqi Lu, Sumei Zhang, Jin Wang

**Affiliations:** ^1^Laboratory of Molecular Biology and Department of Biochemistry, Anhui Medical University, Hefei, China; ^2^Shanghai Public Health Clinical Center, Fudan University, Shanghai, China; ^3^Department of General Surgery, Zhongshan Hospital, Fudan University, Shanghai, China; ^4^Department of Molecular Oncology, Okayama University Graduate School of Medicine, Dentistry and Pharmaceutical Sciences, Okayama, Japan

**Keywords:** circRNAs, KIT, PLAT, ETV1, regulatory networks analysis

## Abstract

Gastrointestinal stromal tumours (GISTs) are the most common human sarcomas and are typically located in the stomach or small intestine. Although circular RNAs (circRNAs) reportedly play vital roles in tumour oncogenesis and progression, the molecular basis of the aggressive tumour biology of these circRNAs in GISTs remains unclear. In this study, we applied SBC ceRNA microarrays to screen for tumour-specific circRNA profiles in GISTs and identified that a total of 5,770 circRNAs and 1,815 mRNAs were differentially expressed in GISTs. Three significantly differential circRNAs (circ_0069765, circ_0084097, and circ_0079471) and their host genes (KIT, PLAT, and ETV1) were also verified in 68 pairs of GISTs and adjacent normal gastrointestinal tissues by qRT-PCR. A GIST-specific circRNA-miRNA-mRNA regulatory network analysis demonstrated that the specific KIT-related regulatory networks involved the three circRNAs, the circRNA host genes and three miRNAs (miR-142-5p, miR-144-3p and miR-485-3p), which may be key regulators of GISTs that could serve as molecular biomarkers and potential therapeutic targets for this malignant disease.

## Introduction

As one of the most common non-epithelial neoplasms, gastrointestinal stromal tumours (GISTs) are located in the stomach (55.6%), small intestine (31.8%), colon and rectum (6.0%), and oesophagus and abdominal cavity (6.2%) and have various clinical features ranging from asymptomatic to nonspecific abdominal discomfort, pain, haemorrhage and tumour abdominal (Joensuu et al., 2012); the incidence of GISTs is 10-15 cases per million per year in 19 countries (Soreide et al., 2016). It is not necessary for GIST patients to exhibit liver metastasis or intraperitoneal dissemination to perform an assessment of the tumour risk. However, clinicopathological factors, including the tumour size, mitotic count and anatomical location, were associated with cancer patient survival (Fletcher et al., 2002; Markku Miettinen and Jerzy Lasota, 2006; Joensuu, 2008), and complete surgical resection remains the primary treatment method for localized GISTs (Ho and Blanke, 2011). GISTs can be characterized by the expression of CD117 or PDGFRA protein in neoplastic cells, and a gain-of-function mutation may exist in the type III receptor tyrosine kinase gene (c-KIT or PDGFR-α) at the genetic level (Hirota et al., 1998; Heinrich et al., 2003b). KIT is a receptor tyrosine kinase that is upregulated in the interstitial cells of Cajal, which are pacemakers responsible for digestive movement (Chi et al., 2010). KIT mutations frequently occur in exons 9, 11, 13 and 17 (Heinrich et al., 2003a; Corless et al., 2004) and play a vital role in GIST pathogenesis (Mazur and Clark, 1983; Hirota et al., 1998). In addition, a PDGFR-α mutation, which is present in 1/3 of KIT-negative GIST patients, mainly affects exons 12, 14 and 18 and can induce tyrosine kinase autophosphorylation, activate signalling molecules containing SH2 domains, and initiate various cancer-related pathways (Wozniak et al., 2012).

Additionally, deregulated circular RNAs (circRNAs) were investigated in acute myeloid leukaemia, breast cancer, gastric cancer, and prostate cancer (Patop and Kadener, 2018; Xia et al., 2018) and found to be involved in a variety of tumour-specific progression events, such as proliferation, invasion and metastasis (Li F et al., 2015; Li J et al., 2015; Wilusz, 2017; Yang et al., 2018; Patop and Kadener, 2018). These deregulated circRNAs exhibit cell- or tissue-specific expression, exist in a steady state on tissues, in the cellular nucleus and cytosol, on extracellular exosomes, and in body fluid and may serve as potential biomarkers of cancer (Gao and Zhao, 2018). Several deregulated circRNAs have been reported to contribute to promoting cell proliferation, such as circPVT1 in gastric tumours, circABCB10 in breast tumours and circBANP in colon tumours (Patop and Kadener, 2018). The downregulation of circITCH was also observed in bladder carcinoma, oesophageal squamous cell carcinoma, lung cancer, colon and rectal cancer and hepatocellular carcinoma (Patop and Kadener, 2018). circRNAs, which have a head-to-tail connected ring structure of exon or intron cyclization, are generated from pre-mRNAs (Wilusz, 2018) and play a sponge role by absorbing microRNAs for binding to the miRNAs of target genes, which could be indirectly influenced by circRNAs forming competing endogenous RNA (ceRNA) networks with circRNAs (Kim et al., 2009). The overexpression of circITCH passively modulated diverse tumour cellular processes by binding miR-17 via specific miRNA-binding sites, which had evident effects on the aggressive biological behaviours mediated by the circITCH/miR-17, miR-224/p21, and PTEN axis (Yang et al., 2018). We previously revealed that the differentially expressed circRNAs (circ_0062019 and circ_0057558) and the host gene SLC19A1 of circ_0062019 could be used as potential novel biomarkers of prostate cancer (Xia et al., 2018). However, to note, no altered circRNAs have been reported in GISTs, and we still lack adequate in-depth knowledge about the biological function of circRNAs in GISTs.

In this study, we first analysed the ceRNA expression profile in gastrointestinal stromal tumour using high-throughput circRNA gene microarray and verified the differential circRNAs in GISTs by qRT-PCR. Our exploration of the circRNA-miRNA-mRNA network could help by adding a new dimension to the study of the molecular mechanisms of GISTs and provide new directions for GIST diagnosis and treatment.

## Materials and Methods

### Patients and Specimens

This study included patients with GIST who underwent partial or complete resection between Sept 2012 and Oct 2017 at Shanghai Public Health Clinical Center, Fudan University, China. The study was approved by the Medical Ethics Commission of Shanghai Public Health Clinical Center. All patients had understood all aspects of the informed consent and signed the informed consent forms before undergoing surgeries. During the operation, 68 pairs of GIST and adjacent normal gastrointestinal tissue samples were collected from obvious lesions and the corresponding gastric or intestinal tissues, which were 1–3 centimetre distant from the tumour edge and contained no obvious cancer cells. After removal from the body, the fresh samples were rapidly intensively chilled in liquid nitrogen within 5 min of excision to avoid degradation. Then, the frozen specimens were stored in a −80°C refrigerator. All enrolled patients were diagnosed for the first time through a pathological examination before undergoing surgical resection. The definitive diagnosis of all cases required tissue biopsy, which relied on endoscopic ultrasound-guided fine-needle aspiration. The tumour histological grading were based on malignancy risk stratification of the gastrointestinal stromal cell tumours by tumour size, mitotic count, and location (Markku Miettinen and Jerzy Lasota, 2006).

### Cell Line, Plasmid and Cell Transfection

The human gastrointestinal stromal tumour cell lines GIST-T1 and GIST-882 were obtained from the American Type Culture Collection (ATCC, Manassas, VA, USA). The GIST-T1 cells were cultured in Mcoy5A’s medium, and the GIST-882 cells were cultured in Dulbecco’s modified Eagle medium (DMEM) supplemented with 10% (v/v) foetal bovine serum (FBS) (HyClone, Logan, UT, USA) under the culture conditions of 37°C and 5% CO_2_. A circ_0084097 and an NC control pLCDH-ciR empty vector were synthesized by Geneseed Biotech Co. Ltd. (Guangzhou, China) and transfected into the GIST-T1 cells by using Lipofectamine 2000 reagent (Life Technologies Corporation, Carlsbad, CA, USA) following the manufacturer’s protocol. The transfection efficiency was assessed using qRT-PCR.

### RNA Purification and SBC ceRNA Microarrays

The total RNA was isolated and purified with TRIzol reagent (Invitrogen, Carlsbad, CA, USA) and a TIANGEN total RNA Isolation Kit (TIANGEN, Beijing, China) according to the manufacturer’s protocol. The isolated RNAs were stored at −80°C. The RNA was qualified, and the RNA integrity number was determined by an Agilent 2100 bioanalyser, while the RNA concentration was analysed using a NanoDrop-2000 spectrophotometer (NanoDrop, USA). For the ceRNA microarray, the included RNA samples were obtained from 3 pairs of GIST and adjacent normal gastrointestinal tissue samples. cRNA was synthesized and amplified with an Agilent Low Input Quick Amp WT Labeling Kit (Santa Clara, CA, US) and can be labelled by cyanine 3-labelled CTP with T7 RNA polymerase. The labelled cRNA was purified by an RNeasy mini kit (Qiagen, USA) and loaded onto SBC Human (4*180K) ceRNA microarrays including 88,371 circRNAs and 18,853 mRNAs (Shanghai Biotechnology corporation, Shanghai, China). The signals were scanned by an Agilent G2565CA Microarray Scanner. The raw data were obtained by Agilent Feature Extraction (v10.7). After normalization of the raw data with R software, the differentially expressed mRNAs and circRNAs were filtrated according to the fold change and Student t-test. The normalized signal value is the value calculated by log2. All ceRNAs with a fold change (FC) ≥ ± 2, a p-value < 0.05 and intensity > 7.0 were included for further statistical analysis. The complete ceRNA array datasets were deposited in the Gene Expression Omnibus (GEO) database under accession number GSE131481.

### Regulatory Network and Pathway Analysis of the Differential mRNAs and the Host Genes of the Differential circRNAs in GISTs

To further investigate the functions of these differential mRNAs in GISTs, the functions of the differential genes were annotated with GO and KEGG pathway analyses (Xia et al., 2018). CircInteractome (https://circinteractome.nia.nih.gov/) was used to predict the putative miRNAs of the three circRNAs and the potential circRNA/miRNA interaction (Dudekula et al., 2016). Targetscan7.2 (https://circinteractome.org./vert_72) was used to predict the targeted miRNAs of the three host genes. We overlapped the two predicted results. Finally, we selected the top miRNAs with the highest context scores (score >85) to establish a circRNA-miRNA-host gene network, which was illustrated by Cytoscape3.5.

### Quantitative Real-Time Polymerase Chain Reaction (qRT-PCR) Analysis of the Differentially Expressed circRNAs and Their Host Genes in GISTs

In total, 3 circRNAs were chosen for experimental validation by qRT-PCR. As an exoribonuclease, RNase R can only act on RNA from its 3’ to 5’ end but does not degrade circRNA (Suzuki et al., 2006). Therefore, to distinguish the expression between the linear mRNA and circRNA, total RNAs were incubated for 20 min at 37°C with or without RNase R (Epicentre Technologies, Madison, WI), and the resulting RNAs were purified using an RNAsimple Total RNA Kit (Tiangen, Beijing, China) and transcribed into cDNA. The cDNAs were synthesized with reverse transcriptase using a PrimeScript^TM^ RT reagent Kit with gDNA Eraser (TaKaRa). The PCR comprised 50 ng cDNA, 10 μl of 2 x PCR Master mix (SYBR Premix Ex Taq^TM^ II kit) (TaKaRa), 0.8 μl primer forward (10 μM), 0.8 μl primer reverse (10 μM), and 0.4 μl of ROX reference Dye and was performed on an ABI ViiA 7 (Applied Biosystems, DE, USA) as follows: denaturation at 95°C for 10 min, amplification at 95°C for 15 s over 40 cycles, followed by annealing and extension at 60°C for 1 min. The results of the relative expression levels were obtained by calculating the raw data using the 2^-ΔΔCt^ method. 18S rRNA served as an internal control for the normalization. The numbers of exons and exact sequences of circ_0084097 produced from PLAT, circ_0069765 from KIT, and circ_0079471 from ETV1 were validated by Sanger sequencing. All the primers for circ_0084097, circ_0069765, and circ_0079471 were designed by Shanghai Biotechnology corporation and shown in **Tables S1** and **S2**.

### Statistical Analysis

To compare the GIST and adjacent normal gastrointestinal tissue samples, the significance of the relative quantification validation was conducted by Student t-test for the paired analysis. All tests were 2-sided, and p < 0.05 was regarded as statistical significance. The data were analysed with Statistical Program for Social Sciences (SPSS) 16.0 software (SPSS, Chicago, IL, USA).

## Results

### Differentially Expressed mRNAs and circRNAs in GISTs

**Figure 1:**
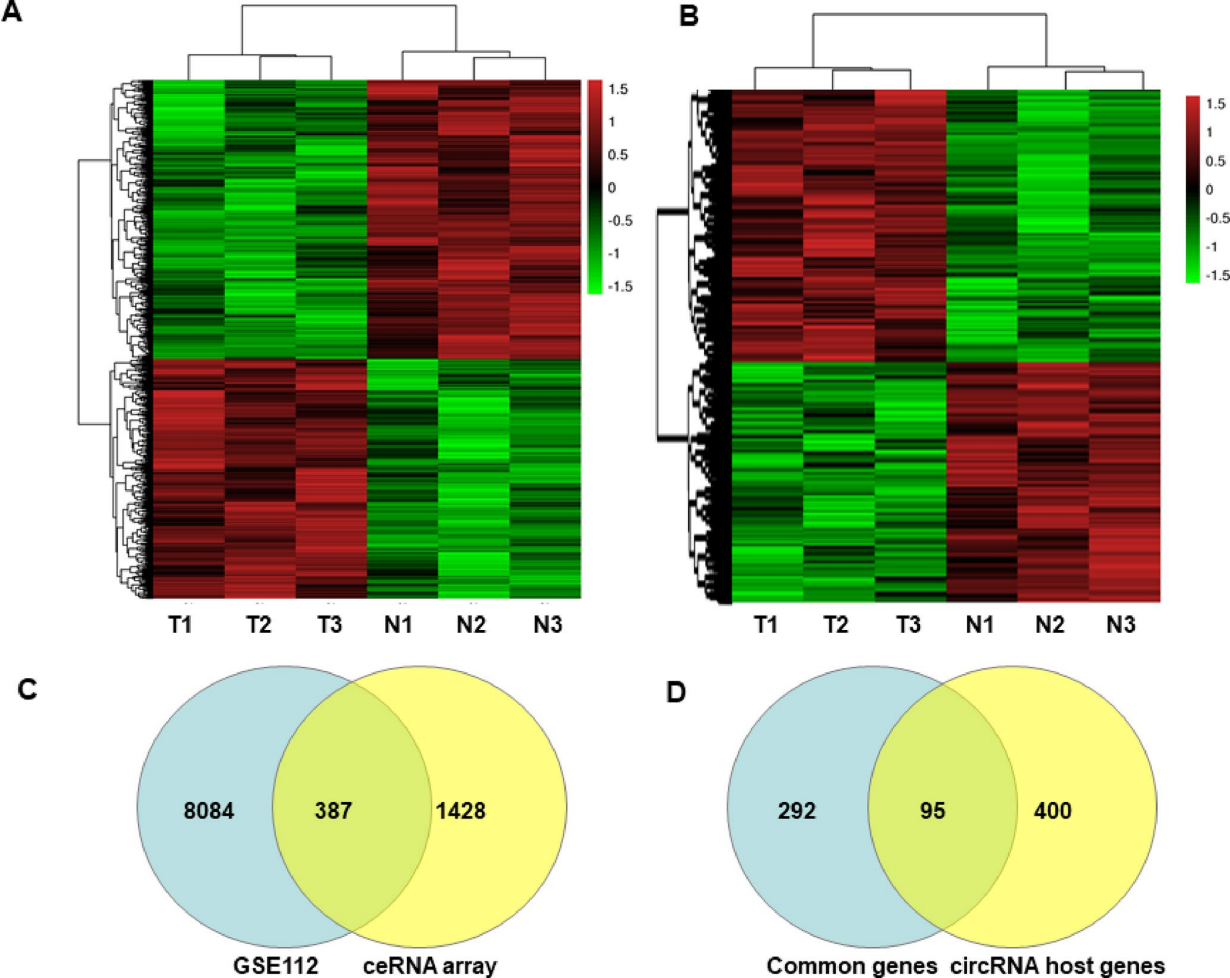
Heatmaps and Venn Diagrams showing the differential mRNAs, circRNAs and their host genes in GISTs. Heat maps of the differentially expressed mRNAs **(A)** and circRNAs **(B)**. Venn Diagrams showing that the common 387 mRNAs **(C)** were from differential mRNAs in the GEO dataset (GSE112) and our ceRNA array, and 95 common mRNAs were from overlapped 387 genes and differently expressed circRNA host genes in our ceRNA array **(D)**.

The characteristics of the GIST patient population and the clinical details of the three samples from the GIST patients chosen for the SBC ceRNA arrays are shown in **Table S3**. The ceRNA arrays were performed to investigate the differentially expressed mRNAs and circRNAs in GISTs. Volcano plots were used to present the significant differences in the extracted data between the GIST and adjacent normal gastrointestinal tissue samples and show the expressed difference in mRNAs (**Figure S1A**) and circRNAs (**Figure S1B**) between the GIST and adjacent tissues. Based on the differences in their expression levels, hierarchical clustering showed the differentially expressed mRNA (**Figure 1A**) and circRNA (**Figure 1B**) expression profile among 3 pairs of GIST and adjacent normal gastrointestinal tissue samples. In total, 1,815 mRNAs (839 upregulated mRNAs and 976 downregulated mRNAs) (**Table 1**) and 5,770 circRNAs (3,122 upregulated circRNAs and 2,648 downregulated circRNAs) (**Table 2**) were differentially expressed between the GIST and adjacent normal gastrointestinal tissue samples (p < 0.05 and FC ≥ ± 2). After screening the differentially expressed mRNAs by retrieving the GEO database (GSE112) and utilizing GEO2R in analysing the array data (**Table S4**), Venn diagrams were generated to show the 387 common differentially expressed genes (DEGs) selected in our array and GEO dataset GSE112 (**Figure 1C**). Finally, 95 DEGs were also identified as the host genes of DEcircRNAs in GISTs. In total, 54 circRNA host genes were upregulated, and 41 DEcircRNA host genes were downregulated in the GIST tumour tissues from these three GISTs patients, which was consistent with the expression level of the circRNAs (p < 0.05 and FC ≥ ± 2) (**Figure 1D** and **Table 3**).

**Table 1:** Partially differentially expressed mRNAs in GIST.

Gene Symbol	Gene bank Accession No	Fold change	*p*-values
MC4R	NM_005912	518.09	0.031
F2RL2	NM_004101	312.59	0.032
LY6H	NM_001130478	205.12	0.008
NPPC	NM_024409	141.15	0.001
FBN2	NM_001999	133.50	0.019
OBSCN	NM_001271223	132.13	0.000
PTPRH	NM_002842	124.36	0.000
ASTN1	NM_004319	112.92	0.027
SCG5	NM_001144757	102.57	0.010
ITGA10	NM_003637	94.89	0.006
TBX5	NM_000192	93.62	0.037
PRKCQ	NM_006257	92.00	0.001
ANO1	NM_018043	84.12	0.001
ABCA12	NM_173076	80.20	0.005
CIT	NM_001206999	63.97	0.000
SH3GL3	NM_001301109	63.28	0.004
KIT	NM_000222	61.06	0.011
DPP10	NM_001178036	57.54	0.018
TENM1	NM_001163278	56.20	0.001
ROBO2	NM_001290040	54.12	0.007
GYG2	NM_003918	−215.06	0.016
PKD1L2	NM_052892	−228.15	0.014
AZGP1	NM_001185	−242.01	0.004
MFAP5	NM_003480	−242.88	0.002
ABCA8	NM_001288985	−258.21	0.002
VIT	NM_053276	−279.41	0.001
CYP4B1	NM_001099772	−281.41	0.004
PI16	NM_153370	−306.57	0.004
HBA1	NM_000558	−315.48	0.008
ITLN1	NM_017625	−335.62	0.004
ALDH1L1	NM_012190	−375.76	0.024
PLIN4	NM_001080400	−392.80	0.009
CFD	NM_001928	−565.13	0.001
C14orf180	NM_001286400	−681.24	0.016
PPP1R1A	NM_006741	−709.72	0.015
HRASLS5	NM_054108	−740.50	0.017
ADH1C	NM_000669	−846.95	0.001
ADH1B	NM_001286650	−886.39	0.021
ADH1A	NM_000667	−969.78	0.003
TUSC5	NM_172367	−2204.07	0.025

**Table 2:** Partially differentially expressed circRNAs in GIST.

circRNA_ID	Gene bank Accession No	Fold change	*p*-values	Host gene
hsa_circ_0065978	NM_001161581	598.50	0.020	POC1A
hsa_circ_0016772	NM_001098623	253.92	0.008	OBSCN
hsa_circ_0016774	NM_001098623	233.44	0.005	OBSCN
hsa_circ_0016775	NM_001098623	231.51	0.004	OBSCN
hsa_circ_0016776	NM_001098623	213.33	0.007	OBSCN
hsa_circ_0016773	NM_001098623	209.56	0.004	OBSCN
hsa_circ_0016778	NM_001098623	201.52	0.004	OBSCN
hsa_circ_0069236	NM_001145847	184.99	0.006	PROM1
hsa_circ_0016780	NM_001098623	180.51	0.001	OBSCN
hsa_circ_0016779	NM_001098623	169.85	0.005	OBSCN
hsa_circ_0023311	NM_018043	137.75	0.001	ANO1
hsa_circ_0028697	NM_001206999	125.79	0.003	CIT
hsa_circ_0028694	NM_001206999	124.24	0.000	CIT
hsa_circ_0028687	NM_001206999	113.21	0.000	CIT
hsa_circ_0045305	NM_138363	107.60	0.000	CEP95
hsa_circ_0073782	NM_001999	103.58	0.023	FBN2
hsa_circ_0023310	NM_018043	103.44	0.000	ANO1
hsa_circ_0003570	NM_014661	98.82	0.000	FAM53B
hsa_circ_0023309	NM_018043	98.25	0.000	ANO1
hsa_circ_0073792	NM_001999	94.47	0.019	FBN2
hsa_circ_0081375	NM_001185	−102.41	0.001	AZGP1
hsa_circ_0037139	NM_000517	−105.37	0.020	HBA2
hsa_circ_0025368	NM_003480	−106.22	0.000	MFAP5
hsa_circ_0035024	NM_001015001	−107.93	0.006	CKMT1A
hsa_circ_0037141	NM_000558	−113.19	0.021	HBA1
hsa_circ_0006751	NM_014241	−115.81	0.003	PTPLA
hsa_circ_0017695	NM_024693	−119.12	0.038	ECHDC3
hsa_circ_0001946	NM_004065	−122.17	0.021	CDR1
hsa_circ_0002091	NM_014241	−130.33	0.000	PTPLA
hsa_circ_0087206	NM_000689	−132.06	0.001	ALDH1A
hsa_circ_0005754	NM_001103184	−146.44	0.022	FMN1
hsa_circ_0080888	NM_006379	−159.28	0.001	SEMA3C
hsa_circ_0025367	NM_003480	−159.49	0.000	MFAP5
hsa_circ_0080897	NM_006379	−159.96	0.001	SEMA3C
hsa_circ_0048861	NM_000064	−181.48	0.004	C3
hsa_circ_0048858	NM_000064	−182.24	0.006	C3
hsa_circ_0048867	NM_000064	−182.38	0.007	C3
hsa_circ_0048892	NM_000064	−196.57	0.010	C3
hsa_circ_0048870	NM_000064	−218.98	0.005	C3
hsa_circ_0048871	NM_000064	−279.58	0.004	C3

**Table 3:** Partial DEcircRNAs and DEGs as host genes in GISTs.

circRNA_ID	Gene bank Accession No	Fold change	*p*-values	Host gene	Fold change	*p*-values
hsa_circ_0073782	NM_001999	103.58	0.023	FBN2	133.50	0.019
hsa_circ_0017609	NM_006257	39.41	0.014	PRKCQ	92.00	0.001
hsa_circ_0028697	NM_001206999	125.79	0.003	CIT	63.97	0.000
hsa_circ_0069765	NM_000222	66.92	0.002	KIT	61.06	0.011
hsa_circ_0056201	NM_001178036	43.76	0.047	DPP10	57.54	0.018
hsa_circ_0091277	NM_198465	70.33	0.000	NRK	51.90	0.000
hsa_circ_0079471	NM_004956	36.48	0.005	ETV1	45.36	0.009
hsa_circ_0008714	NM_001025390	66.38	0.004	AMPD3	33.80	0.003
hsa_circ_0071585	NM_000892	5.39	0.023	KLKB1	24.89	0.026
hsa_circ_0086362	NM_002839	65.77	0.009	PTPRD	23.58	0.009
hsa_circ_0073242	NM_004385	20.36	0.000	VCAN	23.06	0.000
hsa_circ_0084097	NM_000930	19.09	0.013	PLAT	22.19	0.007
hsa_circ_0015753	NM_198503	14.62	0.001	KCNT2	19.19	0.007
hsa_circ_0027663	NM_001135805	29.87	0.001	SYT1	18.99	0.009
hsa_circ_0055922	NM_201555	24.80	0.008	FHL2	16.85	0.000
hsa_circ_0047919	NM_152721	6.70	0.041	DOK6	16.10	0.011
hsa_circ_0052028	NM_002975	16.82	0.003	CLEC11A	16.07	0.003
hsa_circ_0070911	NM_014278	12.28	0.001	HSPA4L	15.89	0.001
hsa_circ_0070487	NM_005723	14.36	0.013	TSPAN5	15.41	0.007
hsa_circ_0069722	NM_145263	29.12	0.002	SPATA18	14.52	0.001
hsa_circ_0080961	NM_000927	25.22	0.019	ABCB1	12.98	0.001
hsa_circ_0015816	NM_205860	−19.14	0.020	NR5A2	−9.17	0.024
hsa_circ_0014229	NM_019554	−41.00	0.008	S100A4	−10.00	0.003
hsa_circ_0033629	NM_001311	−13.51	0.007	CRIP1	−10.04	0.015
hsa_circ_0000895	NM_002229	−8.78	0.009	JUNB	−10.11	0.007
hsa_circ_0070442	NM_007351	−31.47	0.003	MMRN1	−11.11	0.004
hsa_circ_0013276	NM_001013660	−9.10	0.001	FRRS1	−11.70	0.005
hsa_circ_0087214	NM_000700	−38.15	0.033	ANXA1	−12.81	0.007
hsa_circ_0078299	NM_005100	−14.43	0.002	AKAP12	−14.88	0.003
hsa_circ_0060545	NM_002999	−7.21	0.034	SDC4	−16.55	0.016
hsa_circ_0032974	NM_006329	−23.91	0.029	FBLN5	−17.26	0.010
hsa_circ_0000074	NM_002228	−13.85	0.018	JUN	−17.69	0.006
hsa_circ_0055622	NM_207328	−2.59	0.039	GPT2	−18.34	0.002
hsa_circ_0046941	NM_002071	−5.80	0.043	GNAL	−23.67	0.002
hsa_circ_0008591	NM_053025	−18.81	0.025	MYLK	−26.71	0.016
hsa_circ_0049487	NM_001299	−38.06	0.048	CNN1	−28.60	0.041
hsa_circ_0070510	NM_016242	−23.03	0.038	EMCN	−31.55	0.029
hsa_circ_0039466	NM_175617	−11.67	0.001	MT1E	−35.88	0.002
hsa_circ_0056473	NM_032995	−12.44	0.007	ARHGEF4	−45.11	0.000
hsa_circ_0003625	NM_032784	−50.11	0.008	RSPO3	−60.82	0.009
hsa_circ_0025368	NM_003480	−196.57	0.000	MFAP5	−242.90	0.002

### Functional Pathway Analysis of Differential mRNAs and circRNA Host Genes in GISTs

**Figure 2:**
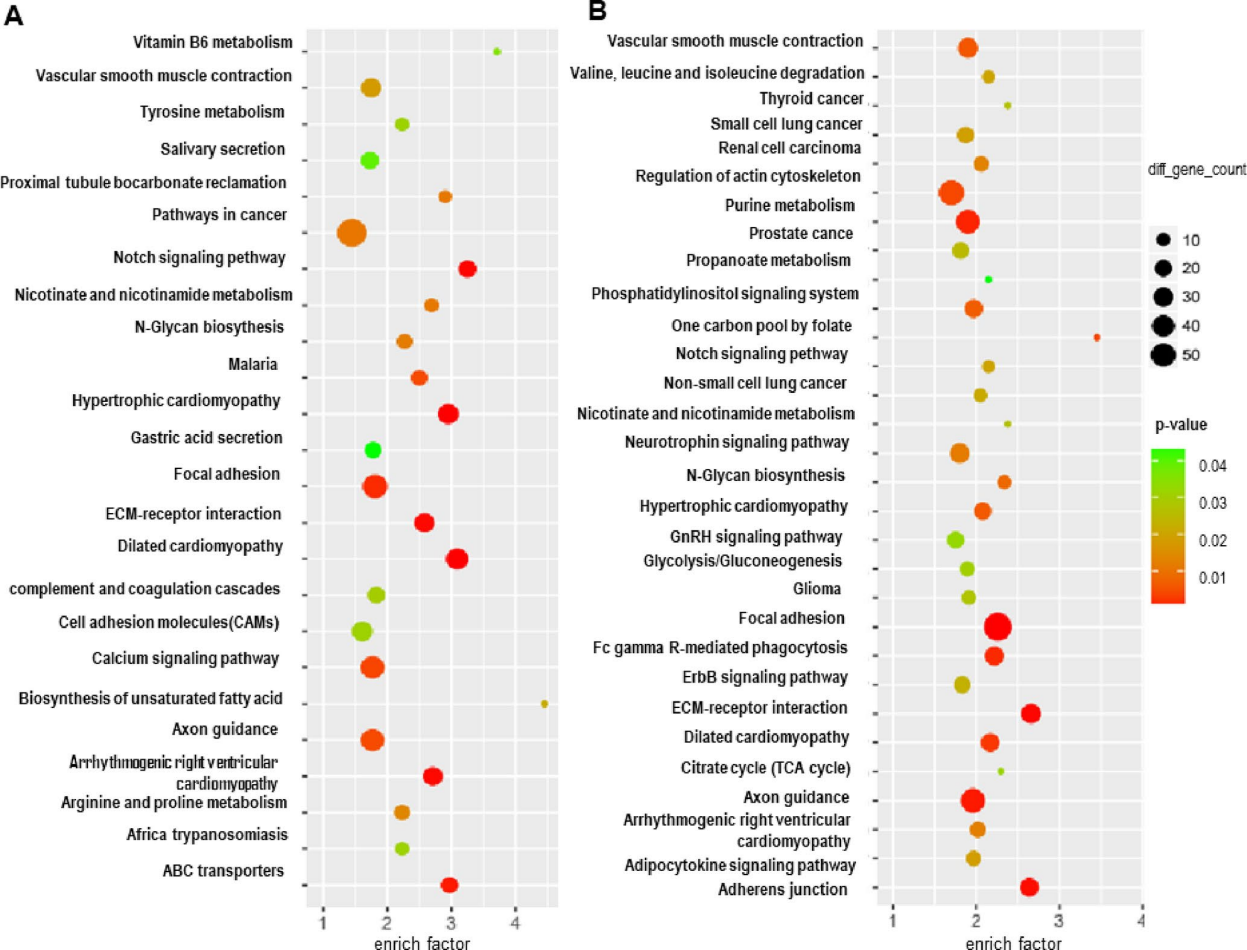
Functional pathway analysis of targeted genes of predicted miRNAs and competitive and endogenous regulatory network. GO analysis of targeted genes **(A)**, and KEGG analysis of targeted genes **(B)**.

Subsequently, a Kyoto Encyclopedia of Genes and Genomes (KEGG) analysis of these differentially expressed mRNAs and the host genes of the differential circRNAs in GISTs was performed to determine the top 5 pathways of the differential mRNAs, which included Biosynthesis of unsaturated fatty acids, Vitamin B6 metabolism, Notch signalling pathway, Dilated cardiomyopathy, ABC transporters and Hypertrophic cardiomyopathy (HCM) (**Figure 2A**); several circRNA host genes were enriched in the pathways of One carbon pool by folate, D-Glutamine and D-glutamate metabolism, ECM-receptor interaction, Adherens junction, and Nicotinate and nicotinamide metabolism (**Figure 2B**). Moreover, several common pathways involved the differentially expressed mRNAs and host genes of differential circRNAs in GISTs, including vascular smooth muscle contraction, Notch signalling pathway, nicotinate and nicotinamide metabolism, N-Glycan biosynthesis, hypertrophic cardiomyopathy (HCM), focal adhesion ECM-receptor interaction, Dilated cardiomyopathy, axon guidance and Arrhythmogenic right ventricular cardiomyopathy (ARVC).

### KIT-Related circRNA-miRNA-mRNA Regulatory Network Analysis in GISTs

**Figure 3:**
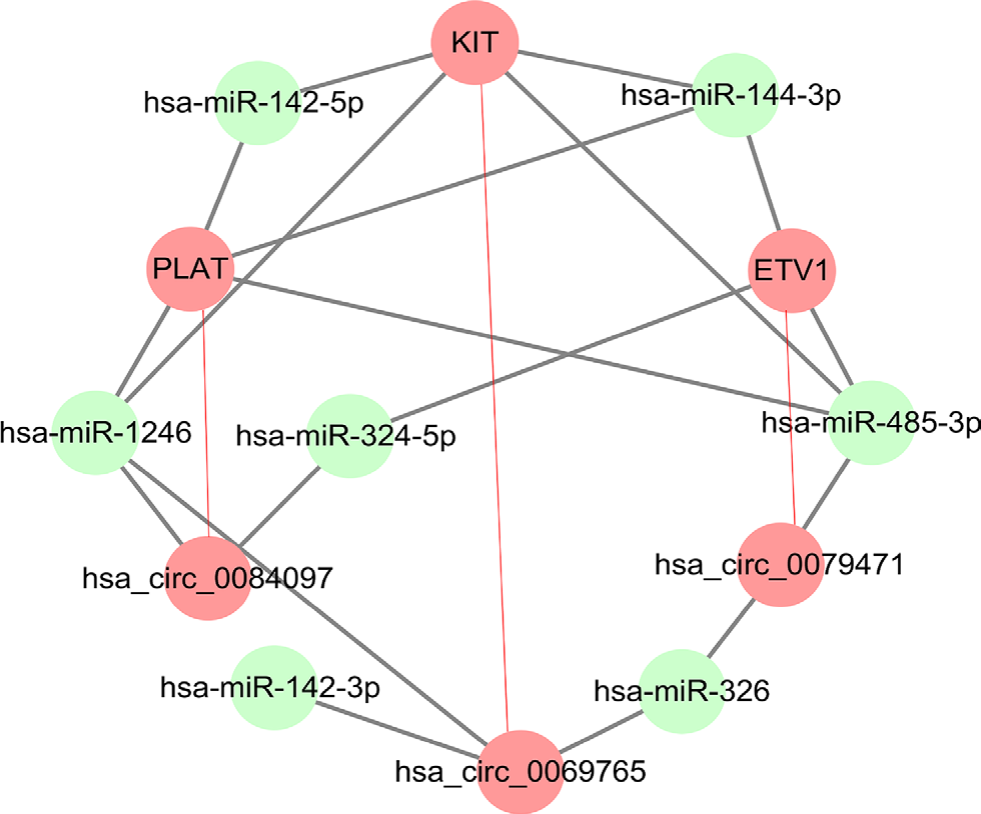
Regulatory network analysis of DEcircRNAs, their targeted genes, and predicted miRNAs. circ_0069765, circ_0079471, circ_0084097 and their host genes (KIT, PLAT, and ETV1) in a related regulatory network.

Among ceRNA expression profiling in GISTs, we found three circRNAs (circ_0069765, cir_0084097, and circ_0079471) and their host genes (KIT, PLAT, and ETV1) were up-regulated in GISTs. The molecular analysis of KIT becomes one of the two gold standards of diagnosis in GISTs. Mutation in the KIT gene is the key oncogenic drivers in the majority of GISTs (Wu et al., 2019), which is also potentiated by a positive feedback circuit that involves the ETS transcription factor ETV1 (Duensing, 2015; Wu et al., 2019). Besides, PLAT (Tissue-Type Plasminogen Activator) as a node with VEGFC, PGF and CHD7 in the functional networks was also verified to be significantly enriched in blood vessel development involved in the tissue specificity of GISTs (Ma et al., 2018), which pushed us to analyze KIT related circRNA-miRNA-mRNA regulatory network in GISTs. Thus, three circRNAs derived from above parental genes were selected for further investigation although there were some top change circRNAs in **Table 2**. circ_0069765, which is located on chr4 q12 (chr4:55569889-55603446), is derived from a non-coding regulatory region of KIT (**Figure S2A**). circ_0079471, which is located on chr7 p21.2 (chr7:13949257-13975521), is a regulatory circRNA within a long non-coding region of ETV1 (**Figure S2B**). However, circ_0084097 stems from a non-coding regulatory region contained a promoter blank adjacent to the promoter region of PLAT, which is located on chr8 p12 (chr8:42046451-42050729) (**Figure S2C**). Based on the miRNA site prediction, we predicted the targeted miRNAs of the three differential circRNAs in circular RNA Interactomem (https://circinteractome.nia.nih.gov/) (Dudekula et al., 2016). To obtain insight into reciprocal circRNA, miRNA and mRNA regulation, we constructed a regulatory circRNA-miRNA-mRNA network using Cytoscape software and clarified the interaction among the three circRNAs (circ_0069765, circ_0084097, and circ_0079471), their host genes (KIT, PLAT, and ETV1) and seven predicted miRNAs (miR-144-3p, miR-1246, miR-485-3p, miR-142-3p, miR-142-5p, miR-326 and miR-324-5p), which is shown in **Figure 3**. In the figure, the upregulated circRNAs and their host genes are marked in red, and the downregulated miRNAs that had been reported in previous studies investigating cancer tissues are marked in green. Evidently, miR-144-3p, and miR-485-3p are common target miRNAs of all three host genes (KIT, PLAT, and ETV1), and miR-142-5p is a targeted miRNA of KIT and PLAT. We also found that miR-1246 was predicted as the common targets of both circ_0069765 and circ_0084097 and their host genes (KIT and PLAT), and miR-326 was predicted as the common targets of both circ_0069765 and circ_0079471. Thus, the specific regulatory networks including the three circRNAs (circ_0069765, cir_0084097, and circ_0079471), their host genes (KIT, PLAT, and ETV1) and the three miRNAs (miR-142-5p, miR-144-3p and miR-485-3p) may be key regulators in GISTs.

### Differential circRNAs (circ_0069765, circ_0079471 and circ_0084097) and Their Host Genes Were Verified in GISTs by qRT-PCR

**Figure 4:**
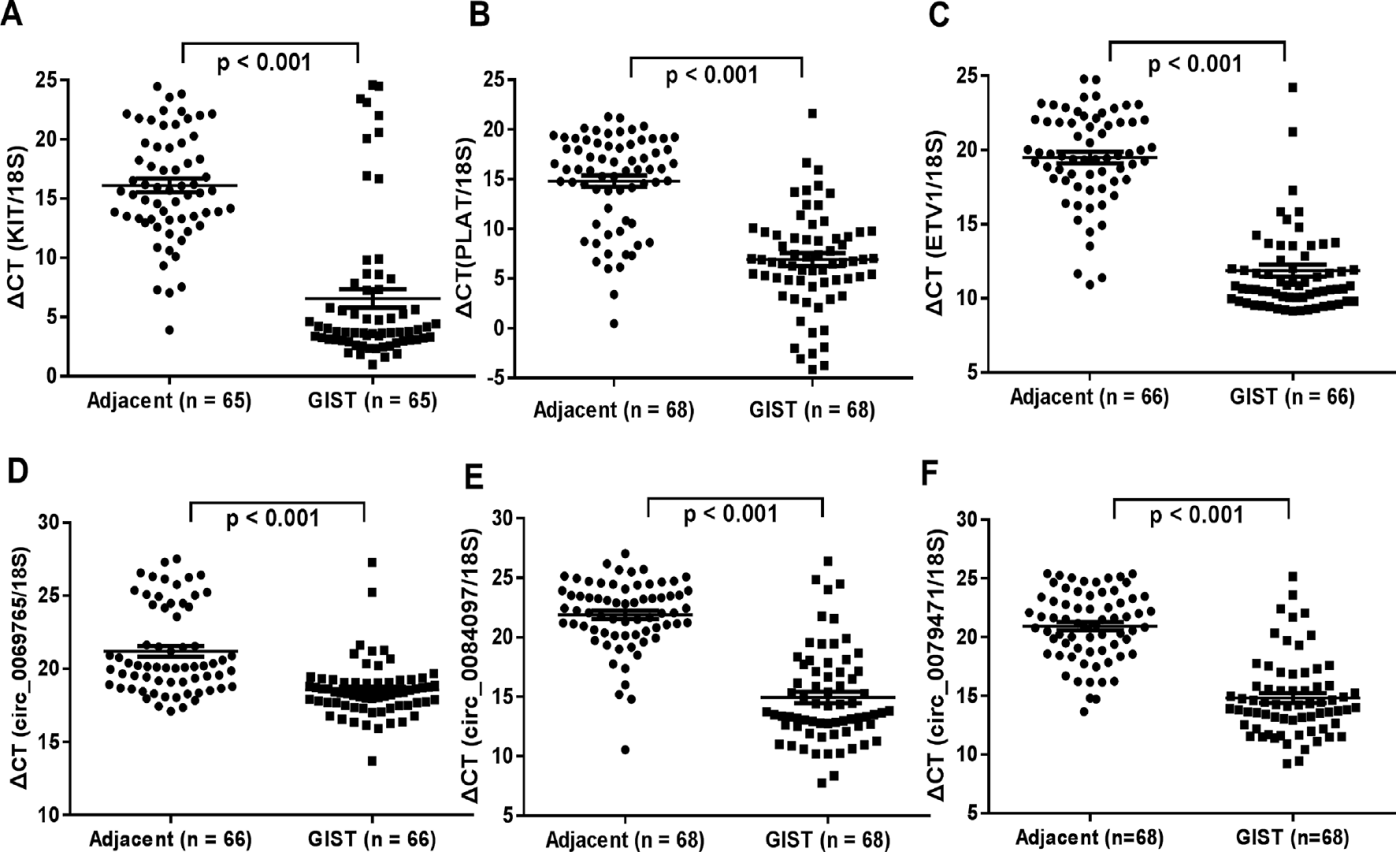
qRT-PCR analysis of the gene expression levels of the three differentially expressed circRNAs and their host genes in GISTs. **(A)** KIT; **(B)** PLAT; **(C)** ETV1; **(D)** circ_0069765; **(E)** circ_0084097; and circ_0079471**(F)**.

The genomic structure shows that circ_0069765 contains six exons from the KIT gene (**Figure S2A**), circ_0079471 contains four exons from ETV1 gene (**Figure S2B**), and circ_0084097 contains three exons from PLAT gene (**Figure S2C**). All the “head-to-tail” splicing sites of the three circRNAs are presented in **Figure S2**. The distinct products of these three circRNAs were amplified using outward-facing primers and confirmed by Sanger sequencing (**Figures S3A–C**). We found that circ_0069765, circ_0079471 and circ_0084097 were resistant to RNase R, compared to the linear mRNAs (Data not shown). Next, we detected the expression level of circ_0069765, circ_0079471, circ_0084097 and their corresponding host genes by real-time PCR (qRT-PCR) analyses. The relative expression of the three circRNAs (circ_0069765, circ_0079471 and circ_0084097) was evidently upregulated in the GIST tissues compared with that in the adjacent noncancerous tissues (p < 0.001); in addition, the three host genes, i.e., KIT, PLAT and ETV1, were upregulated (p < 0.001) (**Figure 4**). The qRT-PCR analyses revealed that 44 of 66 (66.67%) tumours had increased circ_0069765 (4.68-fold); 60 of 65 (92.30%) tumours had increased host gene KIT mRNA (1404.20-fold) expression; 63 of 68 (92.65%) tumours had increased cir_0084097 expression (156.86-fold); 61 of 68 (89.71%) tumours had increased host gene PLAT mRNA (462.43-fold) expression; 59 of 68 (86.76%) tumours had increased circ_0079471 (118.10-fold) expression; and 62 of 66 (93.94%) tumours had increased host gene ETV1 mRNA (678.60-fold) expression. These findings were consistent with the tissue microarray data and showed the significant upregulation tendency of the three circRNAs and three host genes. Finally, we identified that markable positive correlations were present between PLAT and three verified circRNAs (p < 0.05) (**Table S5**). We also noted a non-negative correlation between two circRNAs and ETV1 (**Table S5**, *p < 0.05). Interestingly, an obvious correlation was observed not only between the genes ETV1 and PLAT (p < 0.001) but also between the circRNAs circ_0069765 and circ_0079471 and between circ_0079471 and circ_0084097 (p < 0.05) (**Table S5**). To clarify the characteristics of these differential circRNAs and their host genes in GIST cancer, a Pearson correlation analysis was applied to analyse the correlation between these circRNAs/their host genes and the corresponding clinical parameters. As shown in **Table 4**, circ_0084097 and its host gene PLAT are negatively correlated with metastasis of tumours significantly related to the stomach (p < 0.05). PLAT was also negatively correlated with the tumour diameter (p < 0.05) (**Table 4**), indicating that circ_0084097 and PLAT may be related to the early stage of stomach stromal tumour.

**Table 4:** Correlation analysis of circ_0084097/PLAT expression in tumour tissue samples of GIST patients and their clinical factors.

Characteristic	circ_0084097		Person correlation	p-value	PLAT		Person correlation	p-value
	Low	High			Low	High		
**Location**			0.310	**0.010**			0.284	**0.019**
Stomach	15	24			13	26		
Other	19	10			21	8		
**Metastasis**			−0.246	**0.045**			−0.242	**0.049**
Yes	7	4			8	3		
No	26	30			25	31		
**Vascular invasion**			−0.218	0.074			−0.197	0.108
Yes	7	3			7	3		
No	27	31			27	31		
**Adhesion**			−0.1	0.419			−0.198	0.106
Yes	15	15			19	11		
No	19	19			15	23		
**Maxium tumour diameter**			−0.059	0.635			−0.298	**0.013**
<10 cm	25	28			22	31		
>10 cm	9	6			13	3		
**NIH grade**			0.106	0.388			−0.084	0.496
low risk	15	11			12	14		
intermediate and high risk	19	23			22	20		

Bold values denote statistical significance at the p < 0.05 level.

## Discussion

In this study, the ceRNA expression profile showed that the mRNA and circRNA expression profile in the gastric stromal tumour tissues was distinguished from that in matched tissues adjacent to the tumour and found that a total of 3,122 circRNAs were significantly upregulated and 2,648 were significantly downregulated in the tumour tissues. More importantly, 95 differentially expressed genes had been filtered by overlapping circRNA host genes and significant mRNAs of GSE112. We found several common pathways involving the differential mRNAs and the host genes of differential circRNAs in GISTs, including vascular smooth muscle contraction, Notch signalling pathway, nicotinate and nicotinamide metabolism, N-Glycan biosynthesis, Hypertrophic cardiomyopathy (HCM), Focal adhesion, ECM-receptor interaction, Dilated cardiomyopathy, Axon guidance, and Arrhythmogenic right ventricular cardiomyopathy (ARVC) (**Figure 2**). Three molecular inhibitors of the Wnt signalling pathway have been reported to be tumour suppressors in various in vitro and in vivo GIST models harbouring a KIT mutation. The Wnt antagonist DKK4 was apparently downregulated in advanced human GISTs (Zeng et al., 2017). The Notch signalling pathway has also been reported to be a tumour suppressor in GIST cells harbouring a KIT mutation. The downstream target of notch (dominant-negative Hes1) was apparently upregulated in GIST patients with longer relapse-free survival (Yang et al., 2018). In addition, the focal adhesion signalling pathway played a critical role in the proliferation of both imatinib-sensitive and resistant GIST cells (Zeng et al., 2017). We demonstrated that the Wnt, Notch and Focal adhesion signalling pathways are associated with GIST cell proliferation.

Notably, 95 genes were not only differentially expressed linear RNAs but also maternal genes that generated various differentially expressed circular RNAs in our study. circ_0069765, circ_0079471 and circ_0084097 were selected for the validation of the array results, and we detected the expression of these circRNAs in 68 pairs of tissue samples and showed that the three circRNAs were significantly upregulated in tumour tissues, while their host genes KIT, PLAT and ETV1 had a similar rising trend in expression. Furthermore, the expression levels of these three circRNAs and their host genes were also checked in GIST cell lines. We only found that circ_0069765 was significantly upregulated in the GIST-T1 and GIST-882 cells and that circ_0079471 and its host gene ETV1 were overexpressed in the GIST-T1 cells compared to the normal stomach stromal tissue by a qRT-PCR analysis (all p < 0.05) (**Figure S4**).

**Figure 5:**
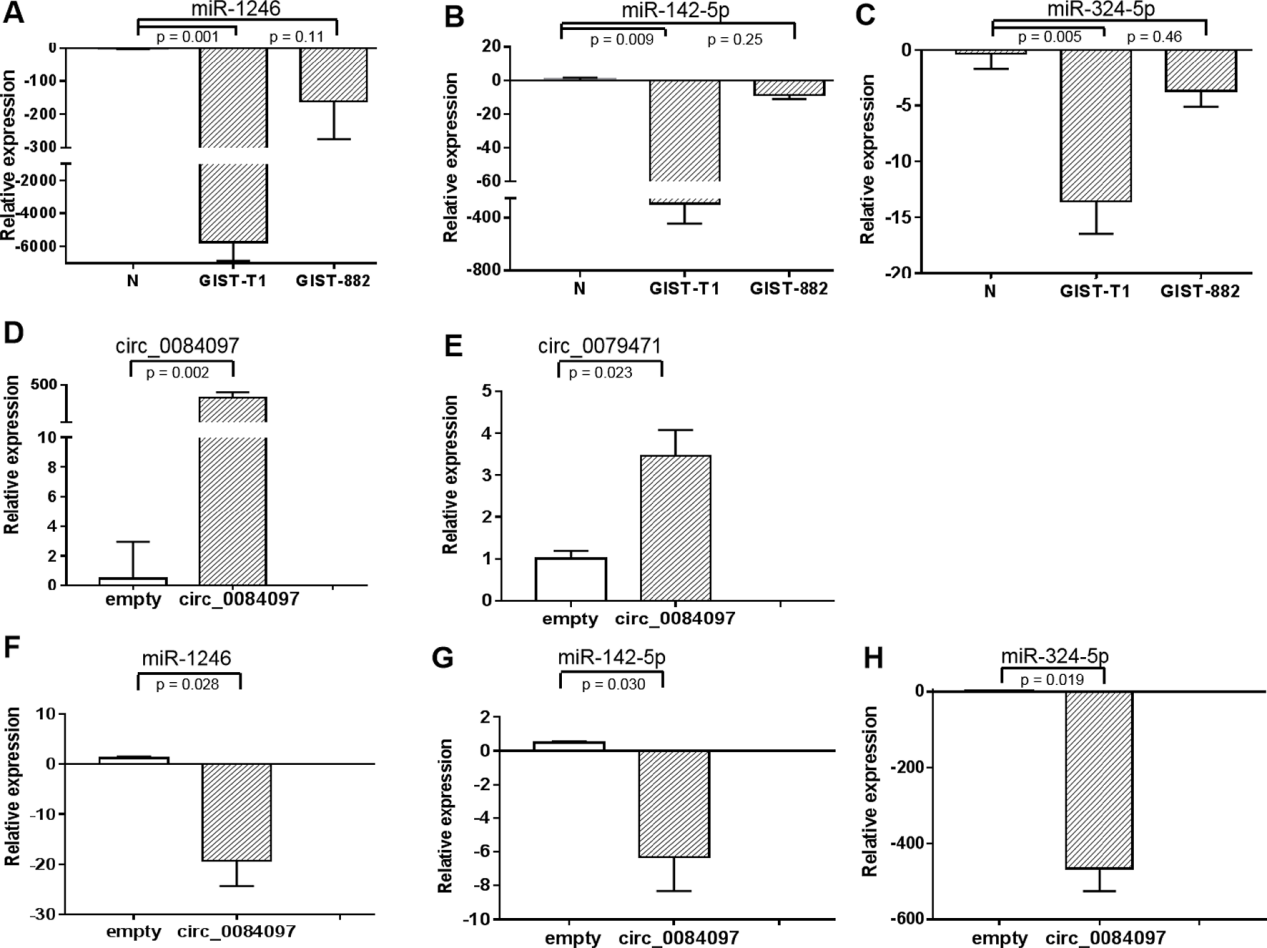
Gene expression levels of miR-1246, miR-142-5p, and miR-324-5p in GIST-T1 and GIST-882 cells **(A**–**C)** **(A)** miR-1246; **(B)** miR-142-5p; **(C)** miR-324-5p and their expression in GIST-T1 with circ_0079471 by overexpression of circ_0084097 **(D**–**H)** **(D)** circ_0084097; **(E)** circ_0079471; **(F)** miR-1246; **(G)** miR-142-5p; **(H)** miR-324-5p were analysed by qRT-PCR.

Web tools for miRNA target-site prediction for circRNA that have a sequence-based recognition system come with the context scores which have the advantage of being predictive for all types of interactions. There is not standard score for selecting top miRNAs. We selected top miRNAs with the high context score (score > 85) for the three differential circRNAs to establish a circRNA-miRNA-host gene network in GIST (**Table S6**) and found that miR-142-3p, miR-142-5p, miR-149, miR-324-5p, miR-326, miR-485-3p and miR-1246 might interact with circ_0069765, circ_0079471 and circ_0084097. Interestingly, miR-1246, miR-142-5p, and miR-324-5p were downregulated in the GIST cells (GIST-882 and GIST-T1) compared to the normal stomach stromal tissue in the qRT-PCR analysis (**Figures 5A–C**). In the analysis of the function of the ceRNAs and their interaction, we confirmed that these three miRNAs were also repressed and that circ_0079471 was upregulated in GIST-T1 cells by overexpression of circ_0084097 (**Figures 5D–H**), which was consistent with our circRNA-miRNA regulatory network analysis in GISTs (**Figure 4**). Thus, these miRNAs may be linked to several host genes, including KIT, PLAT, and ETV1. In GISTs, a KIT proximal domain mutation, especially in exon 11, can induce ligand-independent kinase phosphorylation and activate downstream signal transduction pathway, including AKT, MAPK and STAT (Corless et al., 2011). The molecular targeted agent, Imatinib, blocks KIT / PDGFRA signalling by binding the ATP-binding pocket required for phosphorylation and activation of the receptor. The application of imatinib had changed from a single drug model to a combination with surgical treatment, which was essential to complete surgical resection, alleviate the disease, prolong survival and improve the quality of life, especially among postoperative patients (Huang et al., 2016). Unfortunately, initially sensitive tumours acquired imatinib resistance due to a KIT secondary mutation. Sunitinib and regorafenib are two additional multikinase inhibitors approved as second- and third-line therapies, respectively, and are available for the treatment of imatinib-resistance GIST (Demetri et al., 2006; Demetri et al., 2013). It has been found that non-small cell lung cancer tumourigenesis was suppressed by the overexpression of miR142-5p, which also regulated tumour cell PD-L1 expression and enhanced anti-tumour immunity in pancreatic cancer (Jia et al., 2017; Wang et al., 2017a). The downregulation of miR-142-5p was significantly associated with the recurrence and poor prognosis of gastric cancer (GC) and promoted tumour metastasis by regulating CYR61 expression (Yan et al., 2019). miR-144-3p was significantly downregulated in hepatocellular carcinoma, glioblastoma, multiple myeloma and pancreatic cancer and inhibited proliferation, migration and tumour metastasis by targeting SGK3 (Wu et al., 2017), FZD7 (Cheng et al., 2017), c-Met (Zhao et al., 2017) and FOSB (Liu et al., 2018). The repression of miR-485-3p was also found in breast cancer. The overexpression of miR-485-3p can inhibit mitochondrial respiration and breast cancer cell metastasis by inhibiting PGC-1α expression (Lou et al., 2016). Low serum levels of miR-485-3p were related to poor survival in patients with glioblastoma (Wang et al., 2017b). The miR-324-5p-mediated suppression of NF-κB activation was reported to be responsible for inhibition breast cancer cell invasion and migration (Song et al., 2015). The expression of miR-1246 was downregulated in lung cancer cell lines and cervical cancer tissue, was negatively correlated with the clinical stage and inhibited cell invasion and the EMT by targeting CXCR4 (Yang et al., 2015; Xu et al., 2018). miR-149 was downregulated in ovarian cancer, colorectal cancer and lung cancer. The overexpression of miR-149 increased the drug sensitivity of cancer cells and inhibited the EMT through the FOXM1/cyclin D1/MMP2 axis (Ke et al., 2013; Xu et al., 2015; Sun et al., 2018). Thus, the decreased expression and functional inhibition of these miRNAs in cancer further support our hypothesis that circ_0069765, circ_0079471 and circ_0084097 function to regulate the more comprehensive circRNAs-miRNAs-genes network.

In summary, the present research revealed the ceRNA expression profiles in GISTs and identified that three circRNAs (circ_0069765, circ_0079471 and circ_0084097) and three host genes (KIT, ETV1 and PLAT) were upregulated in GISTs using qRT-PCR. We further demonstrated that the specific regulatory networks including three circRNAs (circ_0069765, cir_0084097, and circ_0079471), their host genes (KIT, PLAT, and ETV1) and three miRNAs (miR-142-5p, miR-144-3p and miR-485-3p) may be key regulators in GISTs and are likely involved in tumour oncogenesis and progression. In future investigations, it is worth considering the verification of the molecular mechanism of these specific circRNAs to regulate GIST occurrence and development. A greater understanding of the mechanisms of the involvement of specific circRNAs in GIST tumour malignancy is necessary for the identification of possible therapeutic targets.

## Data Availability

The datasets generated for this study can be found at NCBI using accession number GSE131481 (https://www.ncbi.nlm.nih.gov/geo/query/acc.cgi?acc=GSE131481).

## Ethics Statement

The study included patients with GIST who underwent partial or complete resection at Shanghai Public Health Clinical Center, Fudan University, China between Sept 2016 and Oct 2017. The study was approved by the Medical Ethics Commission of Shanghai Public Health Clinical Center.

## Author Contributions

JW and HT contributed to the conception; NJ, HT, YZ, HK, YW, WL, SZ, and JW analyzed the data; NJ and JW wrote the manuscript; and JW revised the manuscript.

## Funding

This research was supported by a grant from the National Natural Science Foundation of China (81672383), the National Special Research Program of China for Important Infectious Diseases (2018ZX10302103-003). The grant (KY-GW-2017-09) (HT) was from Shanghai Public Health Clinical Center, Shanghai, China.

## Conflict of Interest Statement

The authors declare that the research was conducted in the absence of any commercial or financial relationships that could be construed as a potential conflict of interest.
