# Supplementary material for: CeRNA Expression Profiling Identifies KIT-Related circRNA-miRNA-mRNA Networks in Gastrointestinal Stromal Tumour
Source: Front Genet. 2019 Sep 10;10:825. doi: 10.3389/fgene.2019.00825 (PMC6746987; doi:10.3389/fgene.2019.00825)

Supplemental Information

CeRNA Expression Profiling Identifies KIT Related circRNAs-miRNA-mRNA Networks in Gastrointestinal Stromal Tumors

Ning Jia[1,,2, #], Hanxing Tong[3, #], Yong Zhang[3, #], Hiroshi Katayama[4], Yuan Wang[1], Weiqi Lu[3], Sumei Zhang[1, *], Jin Wang[2, *]

[1]Laboratory of Molecular Biology and Department of Biochemistry, Anhui Medical University, Hefei, Anhui 230032, China; [2]Shanghai Public Health Clinical Center, Fudan University, 2901 Caolang Road, Jinshan District, Shanghai 201508, China; [3]Department of General Surgery, Zhongshan Hospital, Fudan University, Shanghai 200032, China; [4]Department of Molecular Oncology, Okayama University Graduate School of Medicine, Dentistry and Pharmaceutical Sciences, Okayama, Japan.

Running Title: CircRNAs Expression Profiling Identifies specific circRNAs in GIST

[#] These authors contributed equally to this work

[*]**Corresponding Author:**

Jin Wang, Ph.D.
Shanghai Public Health Clinical Center, Fudan University,
2901 Caolang Road, Jinshan District, Shanghai 201508, China;
Ph: 86-21-37990333-7336;
Fax: 86-21-5724247094
Email: wjncityu@yahoo.com

Sumei Zhang, Ph.D
Laboratory of Molecular Biology and Department of Biochemistry,
Anhui Medical University,
Hefei, Anhui 230032, China
Ph:86-18905699327
E-mail: 379236778@qq.com

1. Supplementary Tables:

1.1) Table S1. The primers for DEcircRNAs and their host genes in GISTs.

Gene/circRNA	Forward Primer	Reverse Primer
KIT	5’- TGTCTGTGTCCAAAGCAAGC-3’	5’- CACCGTGATGCCAGCTATTA-3’
ETV1	5’- CACCGAATTGAGCTCTGTCA-3’	5’- CGAAAGGCTGCAAAAACTTC-3’
PLAT	5’- GGAAGGAGCAAGCCGTGAAT-3’	5’- GCGCAGCCATGACTGATGTT-3’
18s rRNA	5’- GTAACCCGTTGAACCCCATT-3’	5’- CCATCCAATCGGTAGTAGCG-3’
hsa_circ_0069765	5’- CATCCCCACACCCTGTTCAC-3’	5’- CAACGTTGCCTGACGTTCAT-3’
hsa_circ_0079471	5’- CAGGAACCCAGAGATTTTGC-3’	5’- CATTCCCACTTGTGGCTTCT-3’
hsa_cir_0084097	5’- GCCACTCAGTGCCTGTCAAA-3’	5’- CCTCTCTTCATTGCATCCATGA-3’

1.2) Table S2. The primers for miRNAs in GISTs.

Name	Sequence
miR-1246UPLRT	GTTGGCTCTGGTGCAGGGTCCGAGGTATTCGCACCAGAGCCAACCCTGCT
miR-1246FWQ	TCGCGAATGGATTTTTGG
miR-324-5p UPLRT	GTTGGCTCTGGTGCAGGGTCCGAGGTATTCGCACCAGAGCCAACCACCAA
miR-324-5PFWQ	GCGGCGGCGCATCCCCTAGGGCA
miR-142-5p UPLRT	GTTGGCTCTGGTGCAGGGTCCGAGGTATTCGCACCAGAGCCAACAGTAGT
miR-142-5p FWQ	TCGCGCATAAAGTAGAAAGC
RNU6B-UPLRT	GTTGGCTCTGGTGCAGGGTCCGAGGTATTCGCACCAGAGCCAACAAAAATAT
RNU6B-FWQ	TTCCTCCGCAAGGATGACACGC
miRuniRevQ	GTGCAGGGTCCGAGGT

1.3. Table S3. Characteristics of GIST patient population and the clinical detail of the three samples of GIST patients chosen for SBC ceRNA arrays.

characteristic	No. of the case (n = 68)	Percentage (%)	Patient 1 (PID:186795T)	Patient 2 (PID:189169T)	Patient 3 (PID:189776T)
**Age**					
<60	30	44.11	✓ (52)		✓ (55)
>60	38	55.89		✓ (64)	
**sex**					
male	43	63.23			✓
female	25	36.77	✓	✓	
**location**					
stomach	39	57.35	✓	✓	✓
other	29	42.64			
**Metastasis**					
Yes	12	17.64			✓
No	56	82.36	✓	✓	
**Vascular invasion**					
Yes	10	14.7			✓
No	58	82.3	✓	✓	
**Adhesion**					
Yes	30	44.11			✓
No	38	55.89	✓	✓	
**Maxium tumor diameter**					
<10 cm	53	77.94	✓ (4.0)	✓ (5.1)	
>10 cm	15	22.06			✓ (17)
**NIH grade**					
low risk	26	38.23	✓	✓	
intermediate and high risk	42	61.77			✓

1.4) Table S4. Characteristics of the DEGs profiles of GISTs in the GEO datasets.

Accession	mRNA/miRNA	Sample size (tumor/non-neoplastic tissuel)	Platform	Number of assayed genes
GSE112	mRNA	22/7	Human Unigene I	57714
GSE31741	miRNAs	32	Agilent miRNA microarray V3	776

1.5) Table S5. The correlation analysis of the expression of DEcircRNAs and host genes.

**cicRNA/Gene**	**Pearson correlation coefficient**	**P-value**
PLAT/circ_0084097	0.640	0.001
PLAT/circ_0079471	0.701	0.001
PLAT/circ_0069765	0.379	0.003
ETV1/circ_0079471	0.431	0.001
ETV1/circ_0084097	0.242	0.060
ETV1/circ_0069765	0.575	0.000
PLAT/ETV1	0.411	0.001
circ_0069765/circ_0079471	0.424	0.001
circ_0069765/cir_0084097	0.234	0.070
circ_0079471/circ_0084097	0.570	0.000

1.6) Table S6. Context score percentile of 6 miRNAs that bind to the circ_0069765, circ_0084097, and circ_0079471 sequences.

CircRNA/ Mirbase ID	CircRNA - miRNA pairing	Site Type	Context + score percentile
hsa_circ_0069765 (5' ... 3')<br>hsa-miR-1246 (3' ... 5')	UAACAACAAAGAGCAAAUCCAUC ||||||| GGACGAGGUUUUUAGGUAA	7mer-m8	85
hsa_circ_0069765 (5' ... 3')<br>hsa-miR-142-3p (3' ... 5')	UCUUCCCCAUGAUAAACACUACA ||||||| AGGUAUUUCAUCCUUUGUGAUGU	8mer-1a	99
hsa_circ_0069765 (5' ... 3')<br>hsa-miR-326 (3' ... 5')	UGUGGCAGCAGGAUU--CCCAGAGC |||| ||||||| GACCUCCUUCCCGGGUCUCC	7mer-m8	93
hsa_circ_0084097 (5' ... 3')<br>hsa-miR-1246 (3' ... 5')	UUCGCCCAGCCAGGAAAUCCAUG ||||||| GGACGAGGUUUUUAGGUAA	7mer-m8	82
hsa_circ_0084097 (5' ... 3')<br>hsa-miR-149 (3' ... 5')	CCCGAUUCAGAAGAG---GAGCCAGA ||||| ||||||| CCCUCACUUCUGUGCCUCGGUCU	8mer-1a	99
hsa_circ_0084097 (5' ... 3')<br>hsa-miR-324-5p (3' ... 5')	CUGUGAAGCAAUCAUGGAUGCAA |||||| UGUGGUUACGGGAUCCCCUACGC	7mer-1a	88
hsa_circ_0079471 (5' ... 3')<br>hsa-miR-326 (3' ... 5')	AUGAUUAAACAGGAA---CCCAGAGA ||||| ||||||| GACCUCCUUCCCGGGUCUCC	8mer-1a	99
hsa_circ_0079471 (5' ... 3')<br>hsa-miR-485-3p (3' ... 5')	AGUACCACGACCCAGUGUAUGAA ||||||| UCUCUCCUCUCGGCACAUACUG	8mer-1a	93

2. Supplementary Figures:

2.1) Figure S1. Volcano plot showed the DEmRNAs (A) and DecircRNAs (B) in GISTs: blue points represented down regulated mRNA in cancer tissues, while red points indicated up regulated mRNA in tumor samples in volcano plots.

A **B**

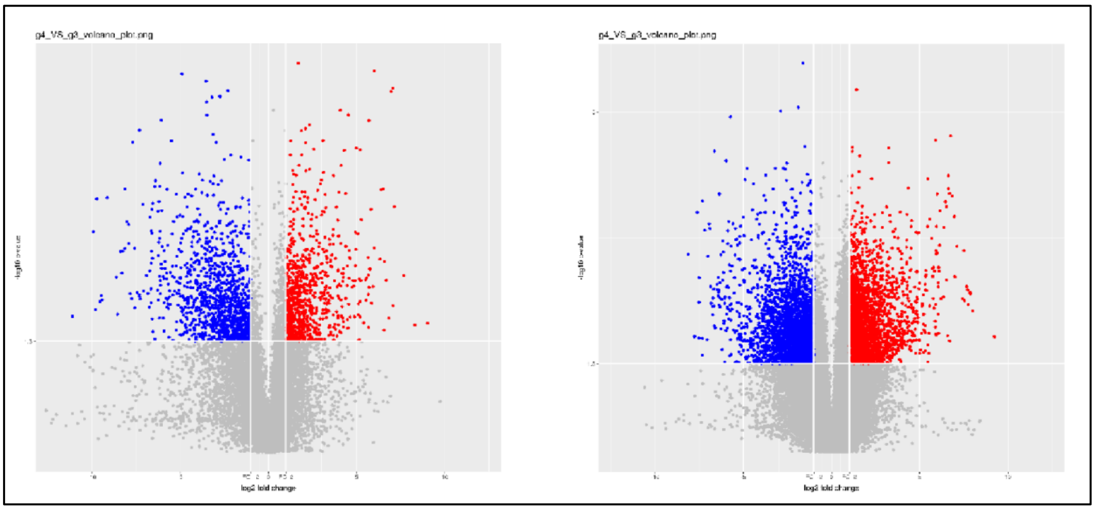

2.2) Figure S2. The location analysis of DEcircRNAs and their host genes in chromosome. A) circ_0069765 and KIT; B) circ_0079471 and ETV1. C) circ_0084097 and PLAT. The distinct products of these three circRNAs were confirmed by Sanger sequencing (Fig. S2D-F). circRNAs were resistant to RNase R, compared to the linear mRNAs (Fig. S2G-I).

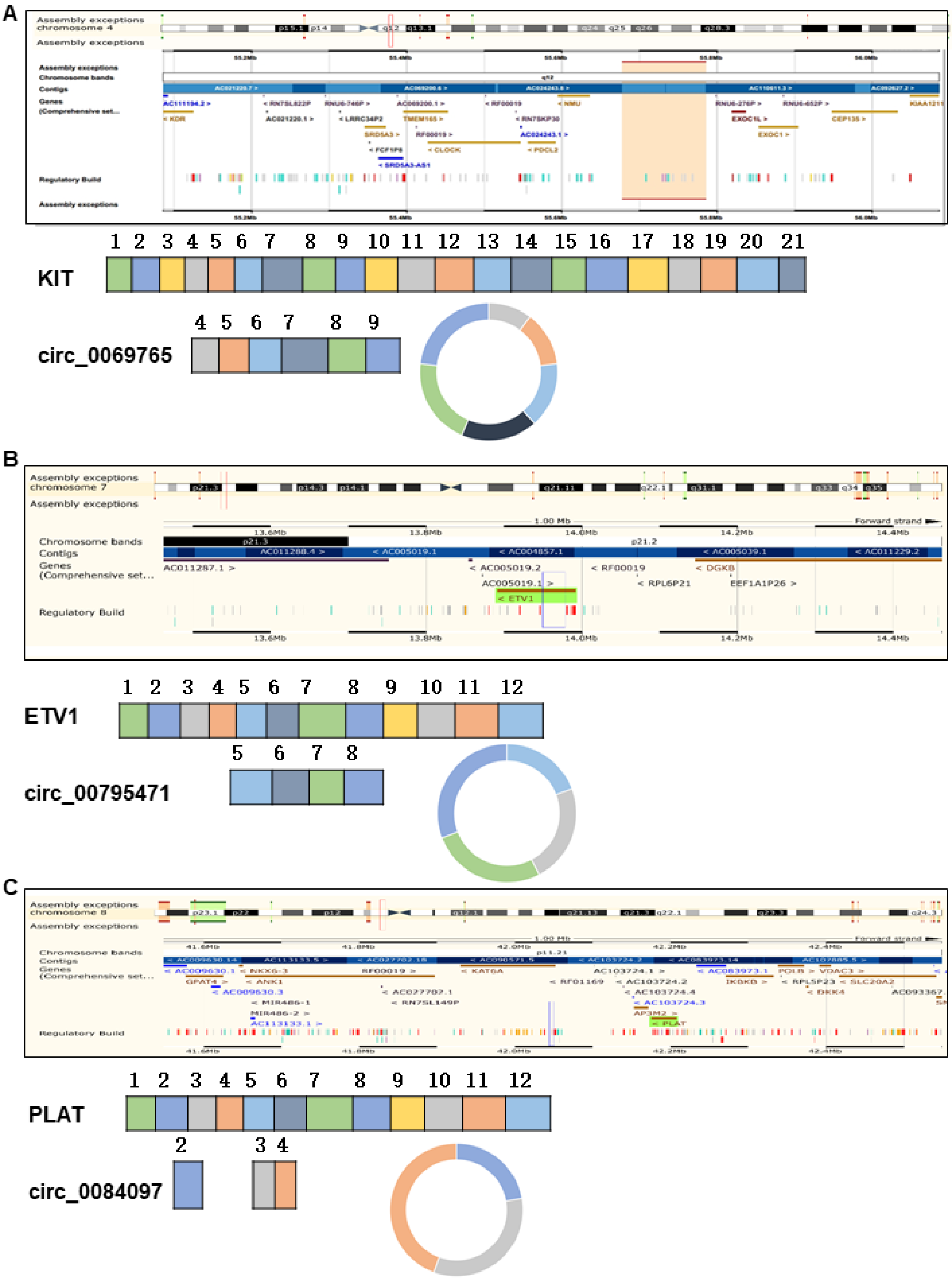

2.3) Figure S3. The sequences of the three circRNAs (circ_0069765, circ_0079471, and cir_0084097) including their splicing junctions were confirmed by Sanger sequencing (A-C): A) circ_0069765; B) circ_0079471; C) circ_0084097; and circRNAs were resistant to RNase R, compared to the linear mRNAs, which were shown in 2% DNA gels (D-F). D) circ_0069765 and KIT; E) circ_0079471 and ETV1. F) circ_0084097 and PLAT.

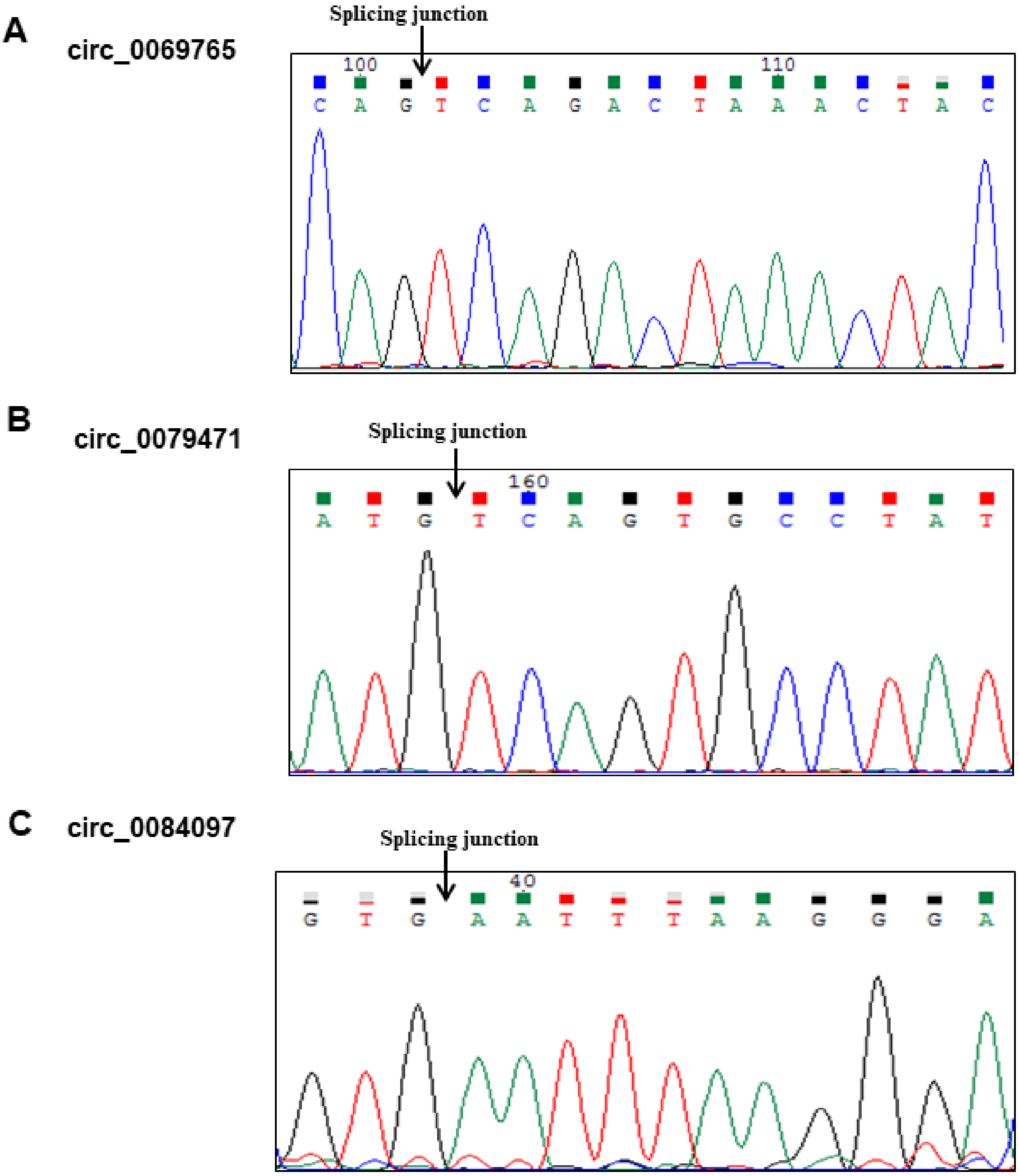

2.4) Figure S4. qRT-PCR analysis of the gene expression levels of circ_0069766, circ_0079471 and ETV1 in GIST cell lines.

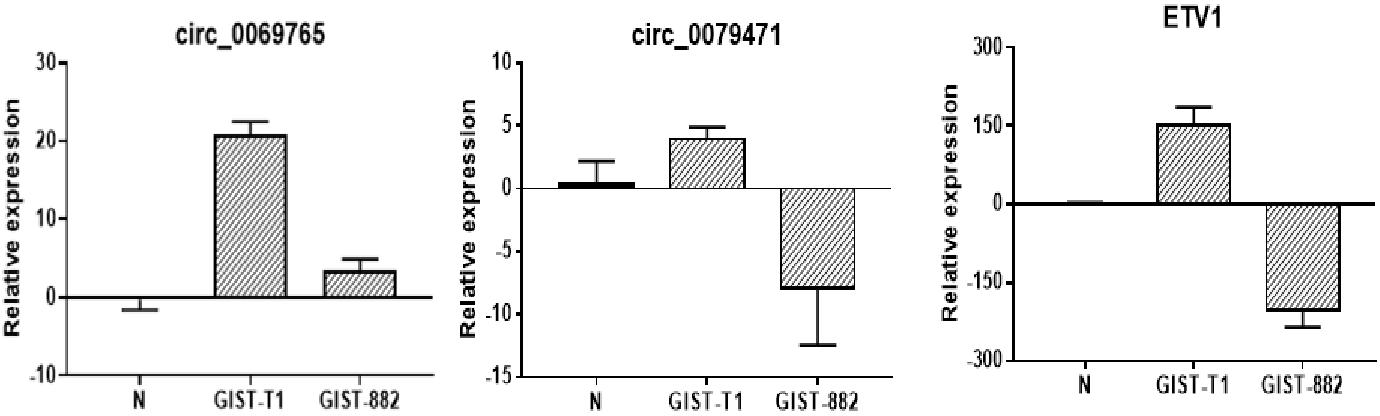

Supplement: Supplementary file 1 [file DataSheet_1.pdf]
